# The purpose of repurposing

**DOI:** 10.18632/oncotarget.14806

**Published:** 2017-01-24

**Authors:** James E. Barrett, Felix J. Kim

**Affiliations:** Department of Pharmacology and Physiology, Drexel University College of Medicine, Philadelphia, PA, USA

**Keywords:** repurposing, polypharmacology, multitarget, mebendazole, trametinib

The goal of developing drugs that act at a single target – the long-standing focus on discovering the ‘magic bullet’ – as enticing and enduring as it has been, has not proven to be a uniformly successful endeavor. Most complex disorders, including those not only in oncology but also in neurology and neuropsychiatry, whether in their initial stages or in their progression, are not mediated by a single protein target. Cancer is a heterogeneous, highly adaptive and constantly evolving disease, driven in large part by the targeted therapeutic agents designed specifically to suppress the disease. The challenges of therapeutic intervention at any one of these stages is likely to necessitate a different strategy which may not only address the existing target(s) but those that emerge following mutations or the development of resistance [1, 2]. While the development of a multitargeted single drug addressing an optimal array of targets poses several challenges, informed drug combinations have the potential to hit multi-sensitive nodes belonging to a network of both static and emerging targets. The repurposing or repositioning of existing drugs that can be used to supplement or enhance therapeutic efficacy is now an active area of research that promises to greatly expand treatment options.

The article by Simbulan-Rosenthal et al. [3] is an important addition to this initiative, elegantly combining a novel rapid computational proteochemometric method [4, 5] coupled to cell-based assays to show that the anthelmintic drug mebendazole should be considered in combination with trametinib as a therapeutic option for patients with NRAS^Q61mut^ or other non-V600E BRAF mutant melanomas.

Mebendazole, discovered in the 1970s, belongs to a class of drugs that bind and disrupt tubulin polymerization. However, mebendazole binds tubulin at a site that differs from other tubulin binding agents such as vinblastine or paclitaxel and, quite remarkably, interacts with a number of other cancer targets. Several of these targets have been identified using the computational methods developed by this group of investigators and include multiple kinases as well as anti-inflammatory drugs [4, 5].

The current study focused mainly on NRAS^mut^/BRAF^WT^ melanoma cells which account for approximately 21% of all melanoma cases and which are particularly recalcitrant to treatment, have shorter survival times than those of patients with melanoma harboring BRAF mutations, and are not responsive to BRAF600 inhibitors such as vemurafinib and dabrafenib. These compounds can actually enhance or inhibit the growth of NRAS^mut^/BRAF^WT^ tumors depending on the cellular context [6]. Consistent with melanoma therapy in general, there have been few treatment options for this genetic cohort.

The computational proteochemometric method developed by Dakshanamurthy et al. [4, 5] used in this report by Simbulan-Rosenthal et al., combines 11 different descriptors that include shape and topology signatures, physiochemical functional descriptors such as docking score and functional contact points of the ligand and target protein, to predict potential drug-target interactions through a method termed “Train, Match, Fit and Streamline” (TMFS). The TMFS method identified mebendazole inhibition of both wild type and V600E mutant BRAF, in addition to other MAPK proteins that include CRAF and MEK. *In vitro* assays confirmed the inhibition by mebendazole in the nanomolar range while also demonstrating an inhibition of both BRAF^V600E^ and BRAF^WT^. The use of structure-based modeling provided further insight by demonstrating the nature of the interactions between mebendazole and BRAF, as well as confirming interactions with VEGFR2 and tubulin. Using two patient-derived melanoma cell lines, BAK and BUL that harbored the BRAF^WT^/NRAS^Q61K^ mutation profile and another melanoma cell line (STU) with a BRAF^600K^/NRAS^WT^ mutation, these investigators demonstrated that mebendazole synergized strongly with trametinib in both BAK and BUL cell lines, but was either antagonistic or additive in STU cells at low or high concentrations, respectively. Further, the combination of these two drugs induced caspase-3 activity earlier and to a greater extent than either drug alone, indicating a rapid induction of apoptosis. Administration of trametinib and mebendazole to nude mice xenografted with BRAF^WT^/NRAS^Q61K^ in BAK cells, resulted in significant inhibition of tumor growth at the higher dose of trametinib and also completely abrogated both MEK1/2 and ERK1/2 phosphorylation. The combination group also remained alive long after animals in the other arms of the study were euthanized.

This study reaffirms the value of examining existing drugs such as mebendazole for potential clinical utility and for treatment options currently less than optimal. The identification of therapeutic need, coupled to sophisticated computational approaches, increasing genetic and genomic information, exemplifies the value of a polypharmacological approach based on repurposing [4, 5, 7] and adds strength to the emphasis on precision oncology and personalized medicine [1, 8]. As this report demonstrates, there is considerable value in examining existing drugs that have been approved or have at least passed through Phase I safety and tolerability studies as the path to clinical application is shortened considerably. It is also clear that even drugs such as mebendazole, on the market for nearly 50 years, still have much to yield in terms of new mechanisms and clinical benefit. Finally, the work by Simbulan-Rosenthal et al. [3] raises the awareness that compound libraries in pharmaceutical companies likely have a large number of “dormant” agents that possess multitarget activity but which were discarded due to ‘off-target’ effects in the quest for selectivity. The computational approach used in this and other studies by these investigators [4, 5, 7] may be a means of extracting valuable information from those libraries and adding new chapters to treatment approaches to oncology and other disorders.
